# Supplementary material for: Extensive co-regulation of neighboring genes complicates the use of eQTLs in target gene prioritization
Source: HGG Adv. 2024 Aug 29;5(4):100348. doi: 10.1016/j.xhgg.2024.100348 (PMC11416642; doi:10.1016/j.xhgg.2024.100348)
Supplement: Document S1. Figures S1–S6, Tables S2–S4, and Note S1 [file mmc1.pdf]

HGGA, Volume *5*

Supplemental information

Extensive co-regulation of neighboring genes complicates the use of eQTLs in target gene prioritization

Ralf Tambets, Anastassia Kolde, Peep Kolberg, Michael I. Love, and Kaur Alasoo

Table of Contents

Figures S1-S6
Tables S2-S4
Supplemental note

Supplemental figures

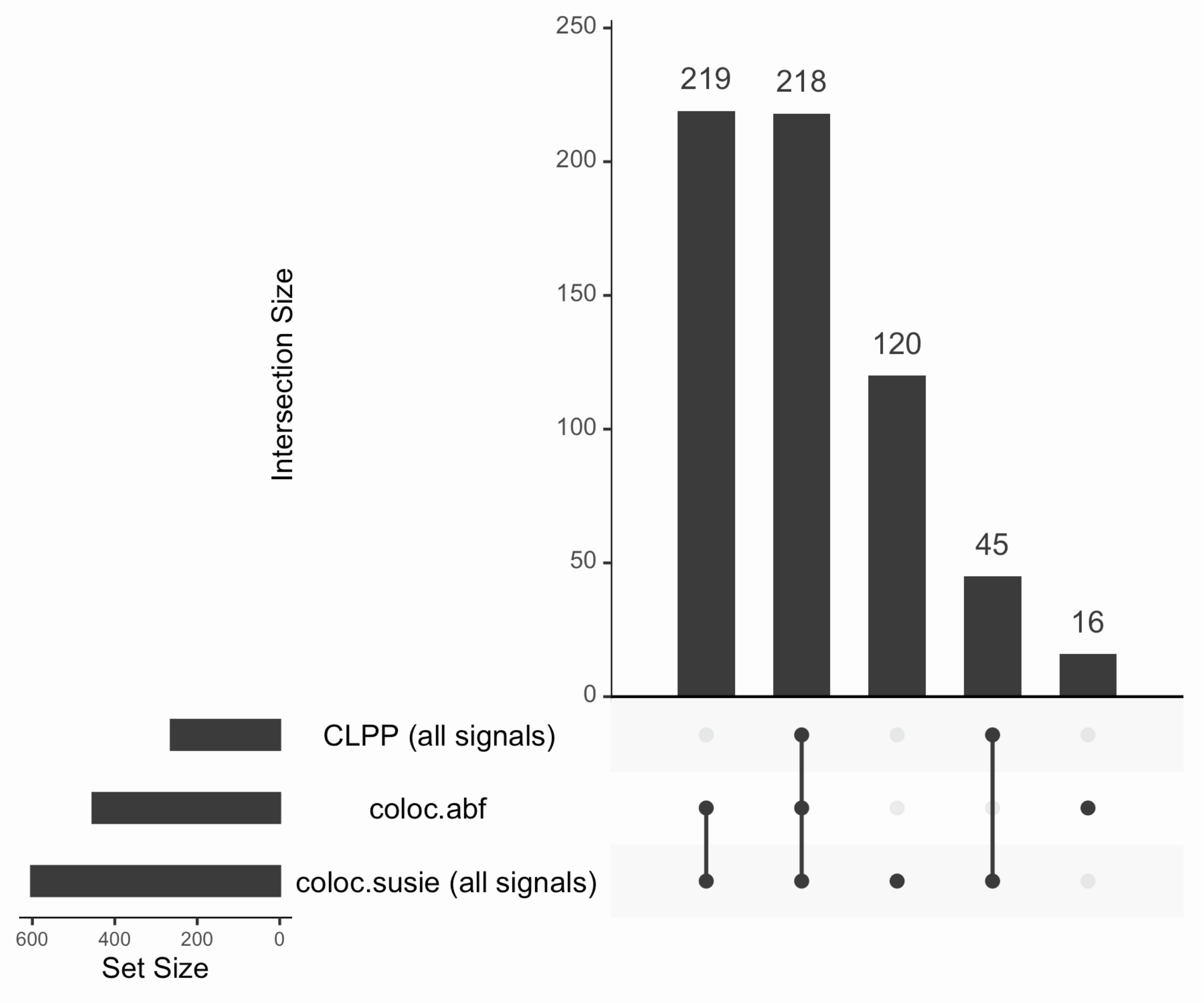

Figure S1. The histogram on the left shows the number of proteins with at least one colocalising eQTL detected by the CLPP, coloc.abf and coloc.susie methods. The histogram on the right is the UpSetR[1] plot showing the overlap of the colocalisation events detected by the three methods. For CLPP and coloc.susie, all fine-mapped pQTL signals were included in the analysis. The total number of proteins included in the analysis was 793.

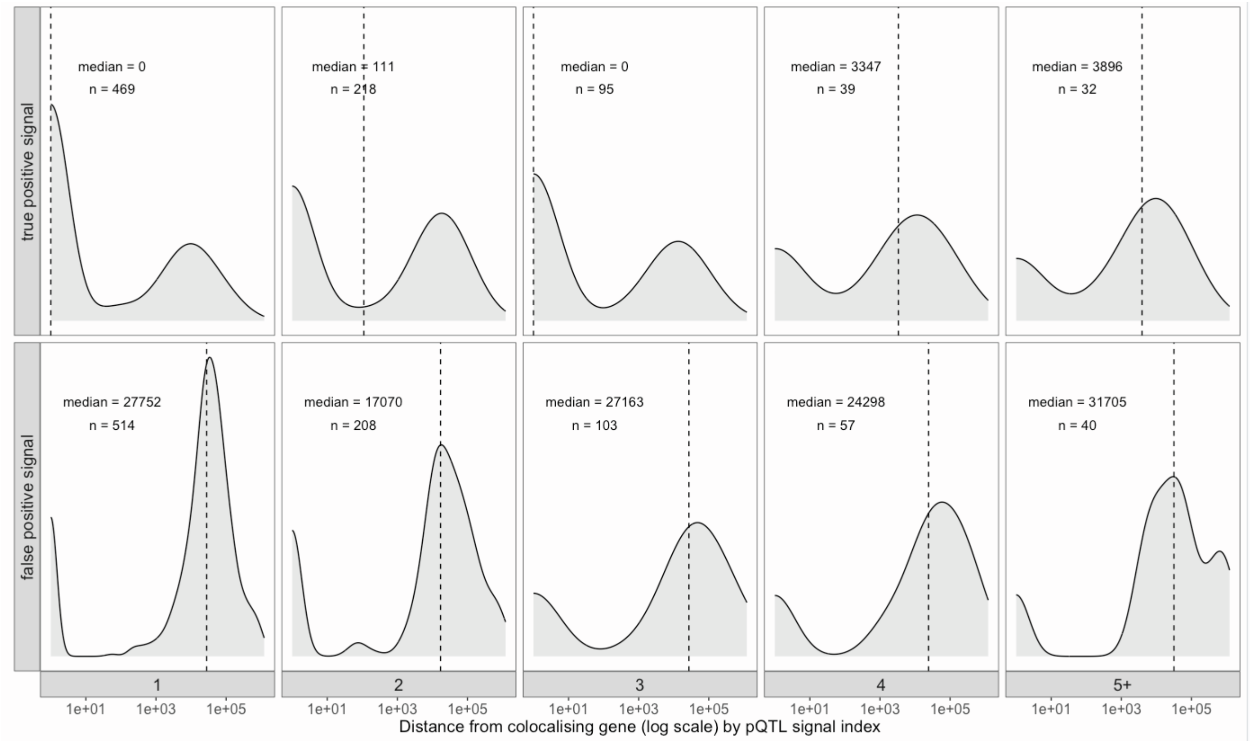

Figure S2. Distribution of distances from the signal-specific fine-mapped lead *cis*-pQTL variant to the corresponding colocalising genes from the coloc.susie analysis. The colocalising genes have been grouped into true positives (top panel) if the gene codes for the protein and false positives (bottom panel) in all other cases. The colocalising signals have also been stratified by the fine-mapped *cis*-pQTL signal index, with primary *cis*-pQTL signals (index = 1) shown on the left.

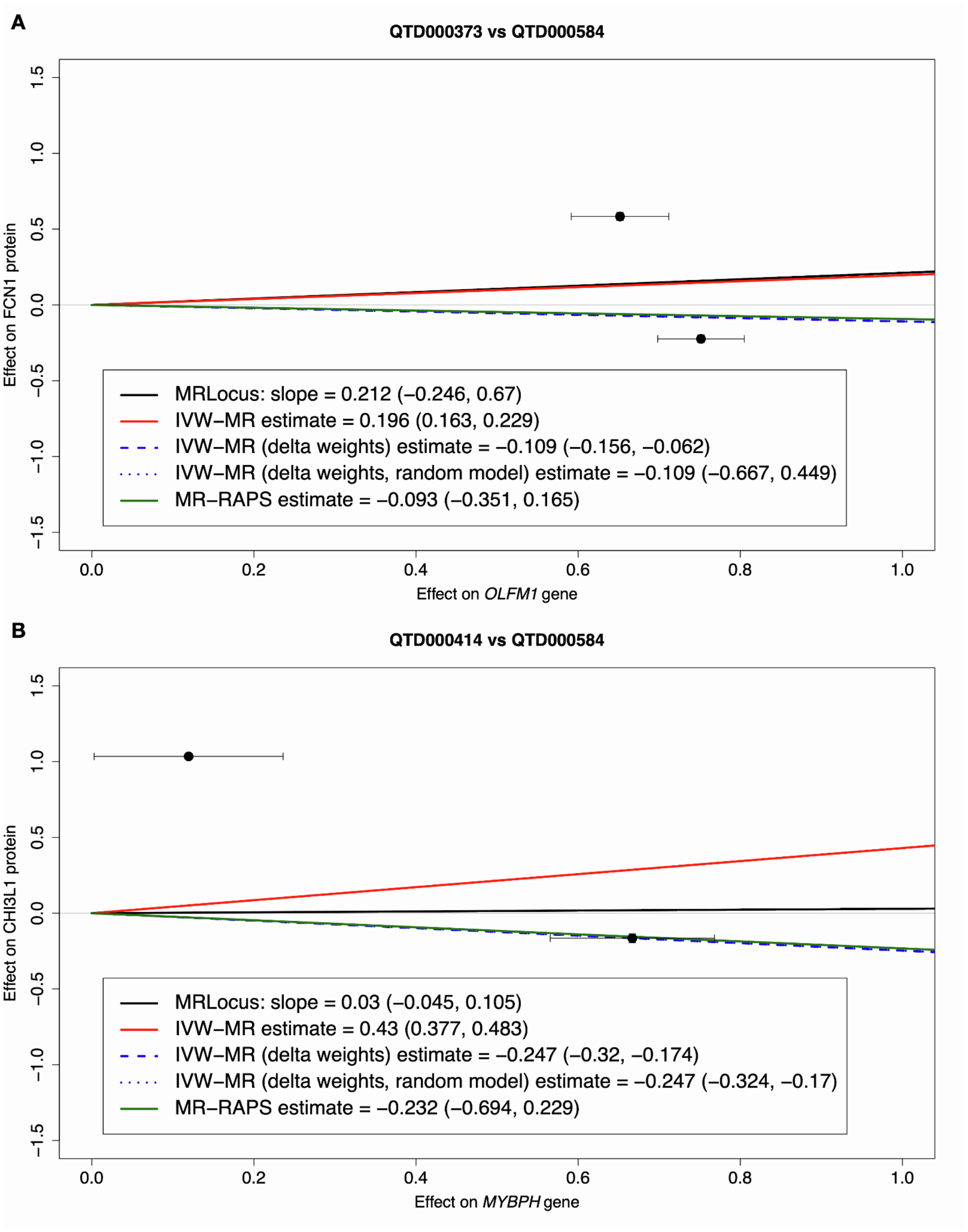

Figure S3. Examples of colocalising eQTLs with discordant effect sizes on protein abundance. (**A**) Discordant effect sizes between olfactomedin 1 (*OLFM1* [MIM: 605366]) gene expression in blood (QTD000373, n = 471) and ficolin 1 (FCN1 [MIM: 601252]) protein abundance in plasma. Bottom panel shows the MR effect size estimates and 80% confidence intervals (80% credible interval for MRLocus) for all five MR methods. (**B**) Discordant QTL effect sizes between myosin-binding protein H (*MYBPH* [MIM: 160795]) gene expression in LPS-stimulated monocytes (QTD000414, n = 184) and chitinase 3-like 1 (CHI3L1 [MIM: 601525]) protein abundance in plasma. Bottom panel shows the MR effect size estimates and 80% confidence intervals (80% credible interval for MRLocus) for all five MR methods

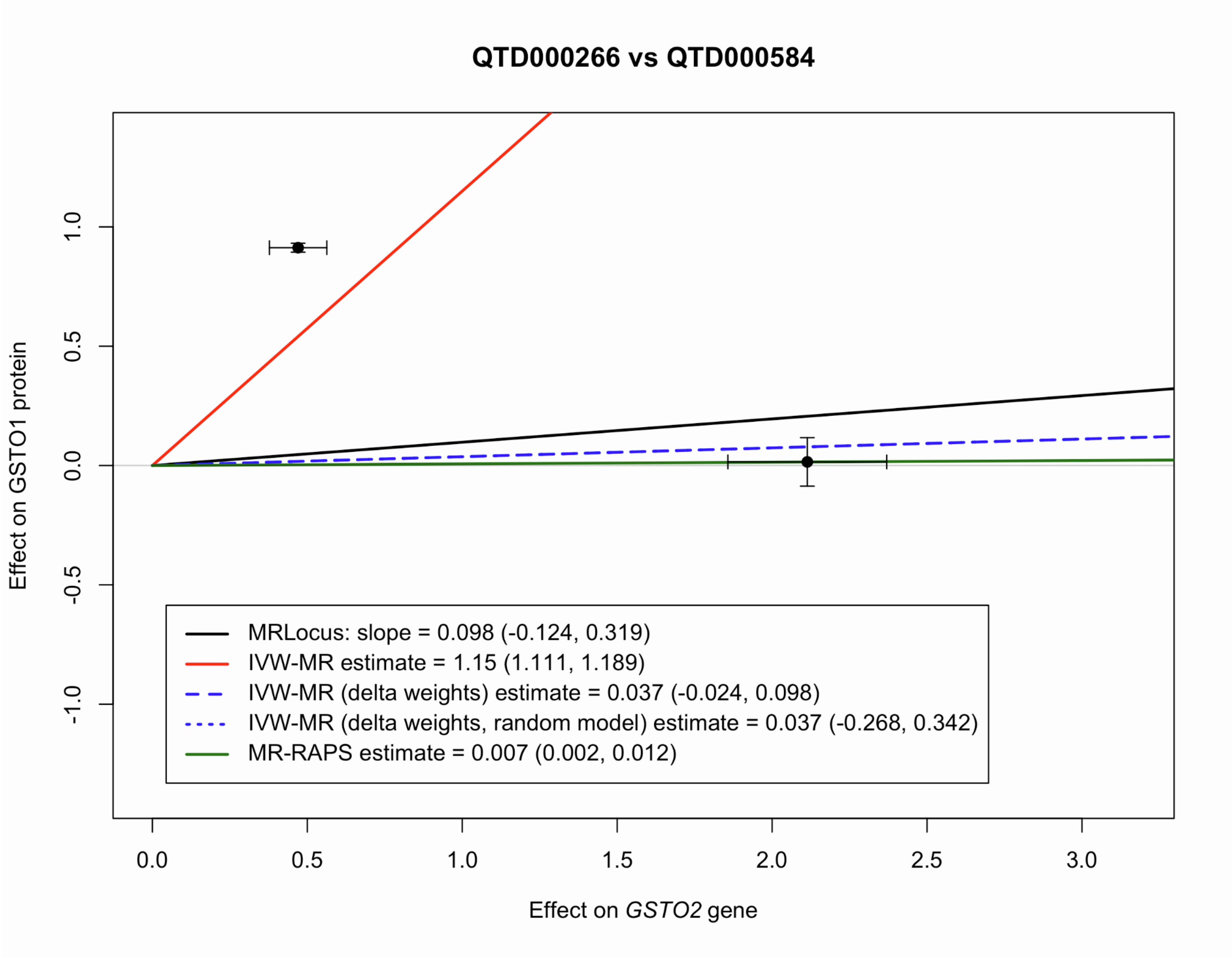

Figure S4. Mendelian randomisation between *GSTO2* gene expression in liver (QTD000266) and GSTO1 protein abundance in plasma using the five MR methods included in the study. IVW-MR infers a very strong positive effect. IVW-MR with delta weights detects a null effect with a narrow 80% confidence interval. Switching to random-effect IVW-MR gives the same causal effect estimate but a much larger confidence interval. This behaviour is also expected from theory.[2] MRLocus gives a similar estimate and 80% credible interval to the random-effect IVW-MR model. MR-RAPS excludes the instrument with a large effect on protein abundance as an outlier and infers a small but very precise effect based on the other instrument alone.

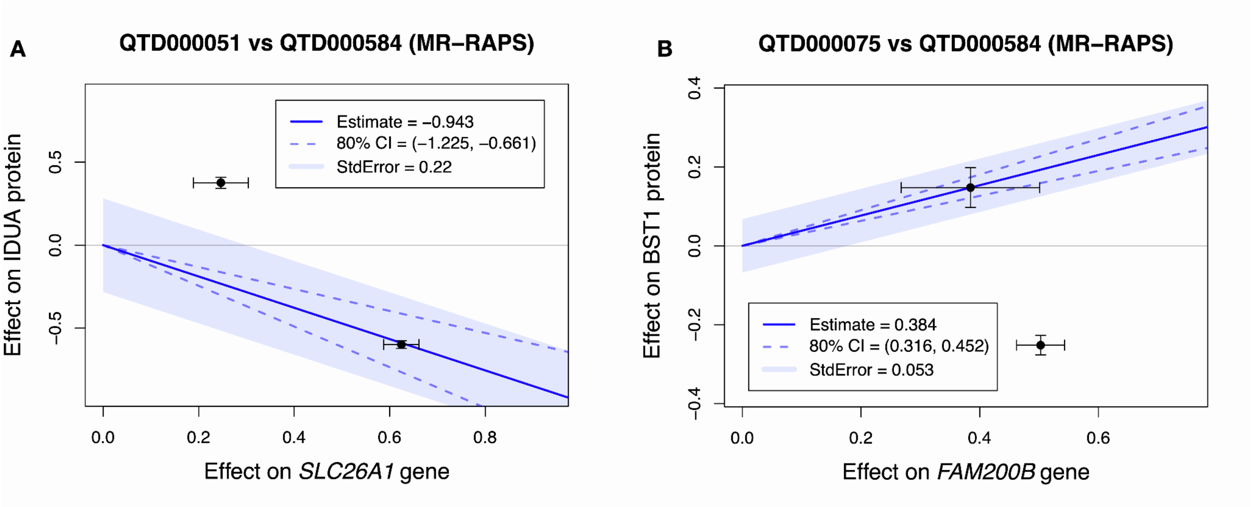

Figure S5. Additional examples of colocalising eQTLs with discordant effect sizes on protein abundance, analysed using MR-RAPS. (**A**) Mendelian randomisation between solute carrier family 26, member 1 (*SLC26A1* [MIM: 610130]) gene expression in brain tissue (QTD000051, n = 479) and alpha-L-iduronidase (IDUA [MIM: 252800]) protein abundance in plasma. (**B**) Mendelian randomisation between *FAM200B* gene expression in ileum tissue (QTD000075, n = 586) and BST1 protein abundance in plasma. In both cases, MR-RAPS discards one variant as an outlier and bases its estimations on the other.

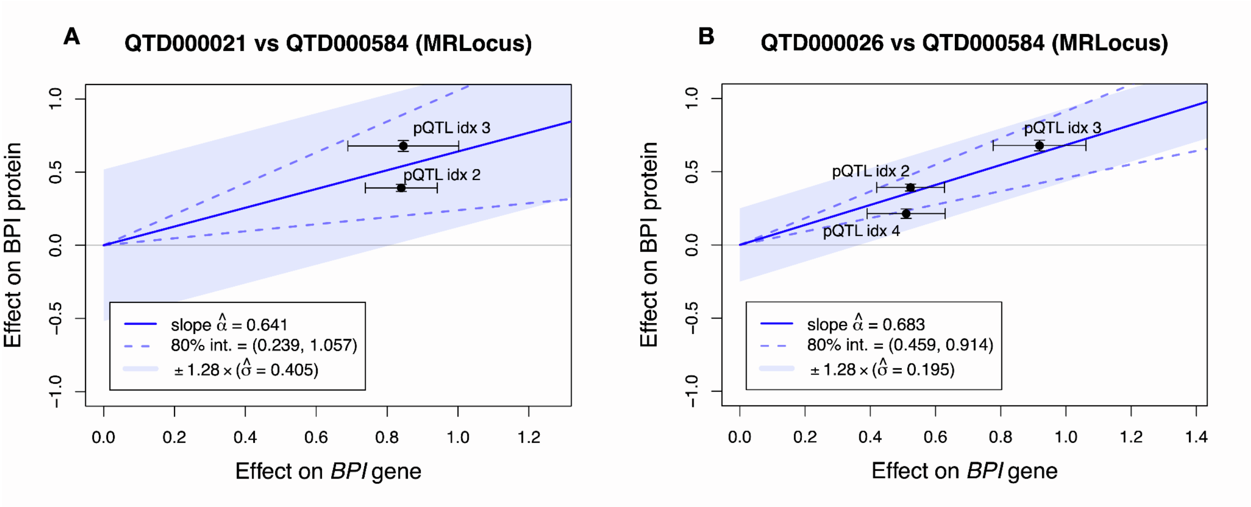

Figure S6. Examples of context-specific changes in colocalisation in the same gene-protein pair. (A) Mendelian randomisation between bactericidal permeability-increasing protein (*BPI* [MIM: 109195]) gene expression in monocytes (QTD000021, n = 191) and BPI protein abundance in plasma. **(B)** Mendelian randomisation between *BPI* gene expression in neutrophils (QTD000026, n = 196) and BPI protein abundance in plasma. Coloc.susie detected an additional independent colocalising signal pair in neutrophils (pQTL idx 4) that was missed in monocytes. Furthermore, although the estimated MR slopes in monocytes (0.641) and neutrophils (0.683) were similar, the allelic heterogeneity estimated by MRLocus was two times smaller in neutrophils compared to monocytes (σ = 0.195 vs σ = 0.405), suggesting that *BPI* gene expression in neutrophils is more likely to causally contribute to BPI abundance in plasma. This is consistent with a much higher neutrophil proportion in whole blood (40%-60%) relative to monocytes (2%-8%).

Supplemental tables

Method	Precision	Recall	TP with positive slope	FP with positive slope
coloc.susie (multiple signals), no MR	68/96 (70.8%)	68/793 (8.6%)	NA	NA
IVW-MR	65/92 (70.7%)	65/793 (8.2%)	238/260 (91.5%)	25/48 (52.1%)
IVW-MR (delta weights)	62/86 (72.1%)	62/793 (7.8%)	230/250 (92.0%)	21/42 (50.0%)
IVW-MR (delta weights, random model)	54/67 (80.6%)	54/793 (6.8%)	201/211 (95.3%)	10/23 (43.5%)
MRLocus	17/19 (**89.5%**)	17/793 (2.1%)	75/75 (**100.0%**)	2/3 (66.7%)
MR-RAPS	62/82 (75.6%)	62/793 (7.8%)	220/241 (91.3%)	15/33 (45.5%)

Table S2. Comparison of the MR methods at 95% confidence interval (95% credible interval for MRLocus). TP with positive slope - the fraction of true positive gene-protein-dataset triplets for which MR fitted a positive slope. FP with positive slope - the fraction of false-positive gene-protein-dataset triplets for which MR fitted a positive slope.

Method	Precision	Recall	TP with positive slope	FP with positive slope
coloc.susie (multiple signals), no MR	29/35 (76.3%)	29/793 (3.7%)	NA	NA
IVW-MR	29/35 (82.9%)	29/793 (3.7%)	28/29 (96.6%)	2/6 (33.3%)
IVW-MR (delta weights)	29/35 (82.9%)	29/793 (3.7%)	28/29 (96.6%)	2/6 (33.3%)
IVW-MR (delta weights, random model)	18/19 (**94.7%**)	18/793 (2.3%)	17/18 (94.4%)	0/1 (0.0%)
MRLocus	20/23 (87.0%)	20/793 (2.5%)	19/20 (95.0%)	1/3 (33.3%)
MR-RAPS	27/31 (87.1%)	27/793 (3.4%)	26/27 (96.3%)	1/4 (25.0%)

Table S3. Comparison of the MR methods on AdipoExpress dataset. TP with positive slope - the fraction of true positive gene-protein-dataset triplets for which MR fitted a positive slope. FP with positive slope - the fraction of false-positive gene-protein-dataset triplets for which MR fitted a positive slope.

Method	Precision	Recall	TP with positive slope	FP with positive slope
coloc.susie (multiple signals), no MR	7/8 (87.5%)	7/793 (0.9%)	NA	NA
IVW-MR	7/8 (87.5%)	7/793 (0.9%)	7/7 (100%)	1/1 (100%)
IVW-MR (delta weights)	7/8 (87.5%)	7/793 (0.9%)	6/7 (85.7%)	1/1 (100%)
IVW-MR (delta weights, random model)	7/8 (87.5%)	7/793 (0.9%)	6/7 (85.7%)	1/1 (100%)
MRLocus	6/7 (85.7%)	6/793 (0.8%)	6/6 (100%)	1/1 (100%)
MR-RAPS	6/7 (85.7%)	6/793 (0.8%)	6/6 (100%)	1/1 (100%)

Table S4. Comparison of the MR methods on TwinsUK adipose dataset. TP with positive slope - the fraction of true positive gene-protein-dataset triplets for which MR fitted a positive slope. FP with positive slope - the fraction of false-positive gene-protein-dataset triplets for which MR fitted a positive slope.

Supplemental note

To investigate the impact of eQTL tissue context on causal gene identification, we quantified the precision and recall of the three colocalisation methods (CLPP, coloc.abf and coloc.susie) in each of the 131 eQTL Catalogue datasets and the AdipoExpress adipose tissue meta-analysis dataset (Table S8). We found that recall depended strongly on the eQTL dataset sample size and was highest in the AdipoExpress dataset (17.8%; coloc.susie; n = 2344), where the corresponding precision was 58.6%. The mean recall for coloc.susie and coloc.abf across all datasets was 3% and mean precision was 67%. For CLPP, the mean recall was 1.2% and the mean precision was 84.5%. To see if restricting colocalisation to trait-relevant eQTL datasets can increase precision, we looked at the GTEx liver (n = 208) and whole blood (n = 670) datasets. In the liver, coloc.susie and coloc.abf methods had 2.1% recall and 52%-55% precision while CLPP had 1.1% recall and 90% precision. In whole blood the recall was higher for all methods (CLPP: 2.4%, coloc.susie: 5.6%, coloc.abf: 5.8%) while precision remained on par with the closest gene approach (CLPP: 70.4%, coloc.susie: 63.8%, coloc.abf = 70.8%). Thus, although restricting eQTL colocalisation to specific datasets can increase precision (e.g. from 45% to an average of 67% for coloc.susie), this is likely driven by limited power of individual eQTL datasets to capture secondary eQTL effects and is also reflected in low average recall (3%).

References

1. Conway, J.R., Lex, A., and Gehlenborg, N. (2017). UpSetR: an R package for the visualization of intersecting sets and their properties. Bioinformatics *33*, 2938–2940.

2. Burgess, S., and Bowden, J. (2015). Integrating summarized data from multiple genetic variants in Mendelian randomization: bias and coverage properties of inverse-variance weighted methods. arXiv [stat.AP].
